# A Rare Case of Meningitis: Can Cellulosimicrobium cellulans Cause Meningitis in a Non-immunocompromised Person?

**DOI:** 10.7759/cureus.72355

**Published:** 2024-10-25

**Authors:** Prajna Narayan, Shishir Duble, Anup Shetty, Mithun Sekhar, Rajesh Shetty, Sathish Kumar Govindarajan

**Affiliations:** 1 Respiratory Medicine, Musgrove Park Hospital, Taunton, GBR; 2 Neurology, First Neuro Hospital, Mangalore, IND; 3 Microbiology, Father Muller Medical College, Mangalore, IND; 4 Neuroradiology, Kasturba Medical College, Manipal, Mangalore, IND; 5 Internal Medicine, Glangwili General Hospital, Carmarthen, GBR

**Keywords:** afebrile, altered sensorium, atypical bacterial meningitis, cerebrospinal fluid (csf), leptomeningeal enhancement, photophobia

## Abstract

Infections with Gram-positive soil-dwelling *Cellulosimicrobium cellulans* bacterium are sporadic. Rarely, do patients with indwelling medical devices or those who suffer from immunosuppression get infected by this pathogen. However, based on routine clinical and laboratory procedures, it is hard to distinguish between the meningitis caused by *C. cellulans* and that from other bacteria. Here, we report a unique case of *C. cellulans* infection in a 37-year-old immunocompetent man presenting with meningitis associated with encephalopathy and headache. He presented with severe headaches, altered sensorium, reduced sleep, photophobia, and restlessness, with a feeling of impending doom, but with no neck rigidity and fever. Trans-axial T1 and T2/FLAIR head MRI showed diffused cerebral edema, with bilateral high frontoparietal sulcal enhancement, hyperintensity along the right posterior insula-temporal region, and left parietal deep white matter. Lumbar puncture CSF examination indicated bacterial meningitis, and *C. cellulans* was identified on culture. The patient was administered intravenous ceftriaxone for seven days and dexamethasone for three days. A follow-up lumbar puncture CSF examination showed no signs of the pathogen, indicating its eradication. To our knowledge, this is the first case of *C. cellulans* causing meningitis in an otherwise healthy man with no history of indwelling medical devices or immunosuppression. This rare case of meningitis suggests that *C. cellulans* can infect healthy humans and cause meningitis.

## Introduction

Bacterial meningitis is a common infectious cause of meningitis. The most common bacteria causing meningitis are respiratory pathogens such as *Streptococcus pneumoniae, Neisseria meningitis, and Hemophilus influenza *[1]. Apart from epidemics, around 1.2 million cases of bacterial meningitis occur each year. According to the World Health Organization Meningitis 2021, around 1 in 10 patients with bacterial meningitis die, and 1 in 5 suffer from severe complications [1]. We report a case of a 37-year-old male diagnosed with meningitis caused by *Cellulosimicrobium cellulans (C. cellulans*), previously identified as *Oerskovia xanthineolytica*, which is a Gram-positive filamentous rod-shaped bacterium found predominantly in soil and water. Infection with *C. cellulans* in humans is rare as noted by Rivero et al. [2]. This case report is unique as this patient had no implanted medical devices and was immunologically adept, as the bacteria invade the human system almost always through implanted medical devices or due to extreme immunosuppression.

This article was previously presented as a meeting abstract at the 2024 British Associations of Physicians of Indian Origin (BAPIO) meeting on September 19, 2024.

## Case presentation

Patient description

A 37-year-old man presented with a two-day-long headache and altered sensorium. The headache started acutely and was throbbing mainly localized to the occipital region associated with photophobia with a feeling of impending doom. However, the headache was not associated with head trauma, vomiting, seizures, or fever. He had no history of similar symptoms in the past, systemic illness, previous hospitalization, any surgical interventions, history of medical device implantation, history of substance abuse, or immunosuppression. He was a manual laborer who did odd jobs, mainly construction work. He was married, monogamous, and had two children.

Investigation results

**Figure 1:**
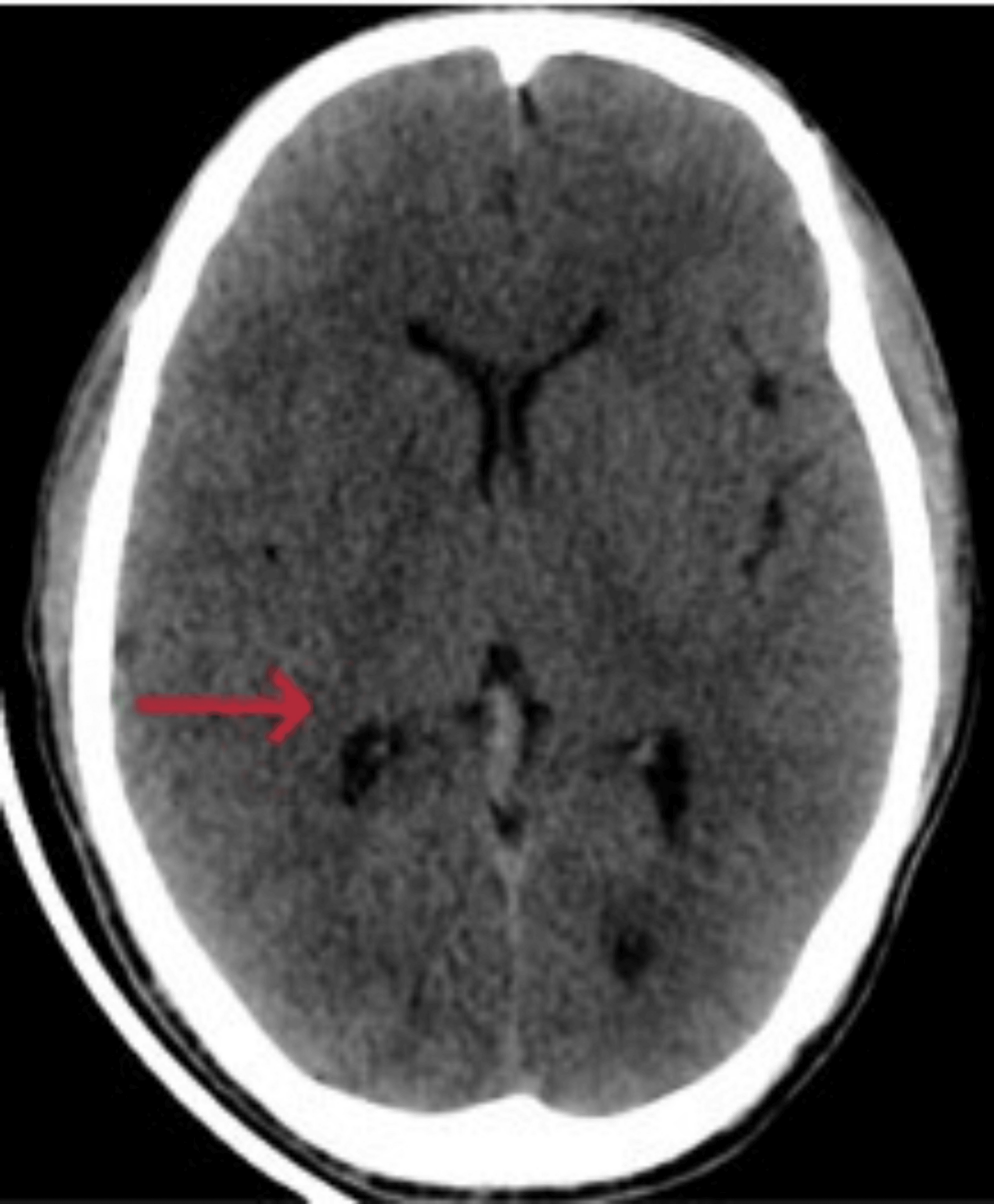
Trans axial CT scans passing through the third ventricle showing diffuse sulcal effacement indicating mild cerebral edema. CT: Computed tomography

**Figure 2:**
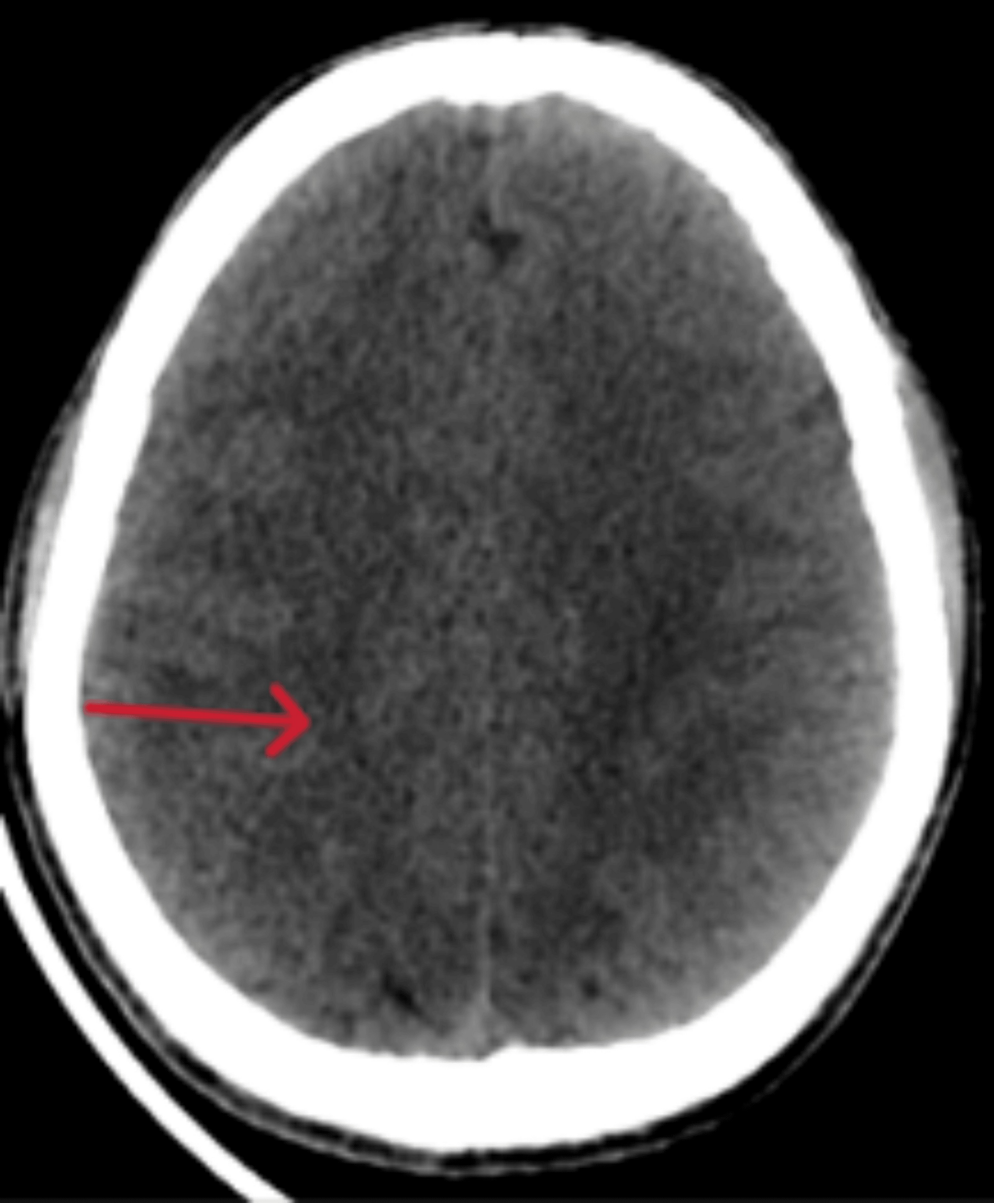
Trans axial CT scans passing through the high parietal cortex showing diffuse sulcal effacement indicating mild cerebral edema. CT: Computed tomography

**Figure 3:**
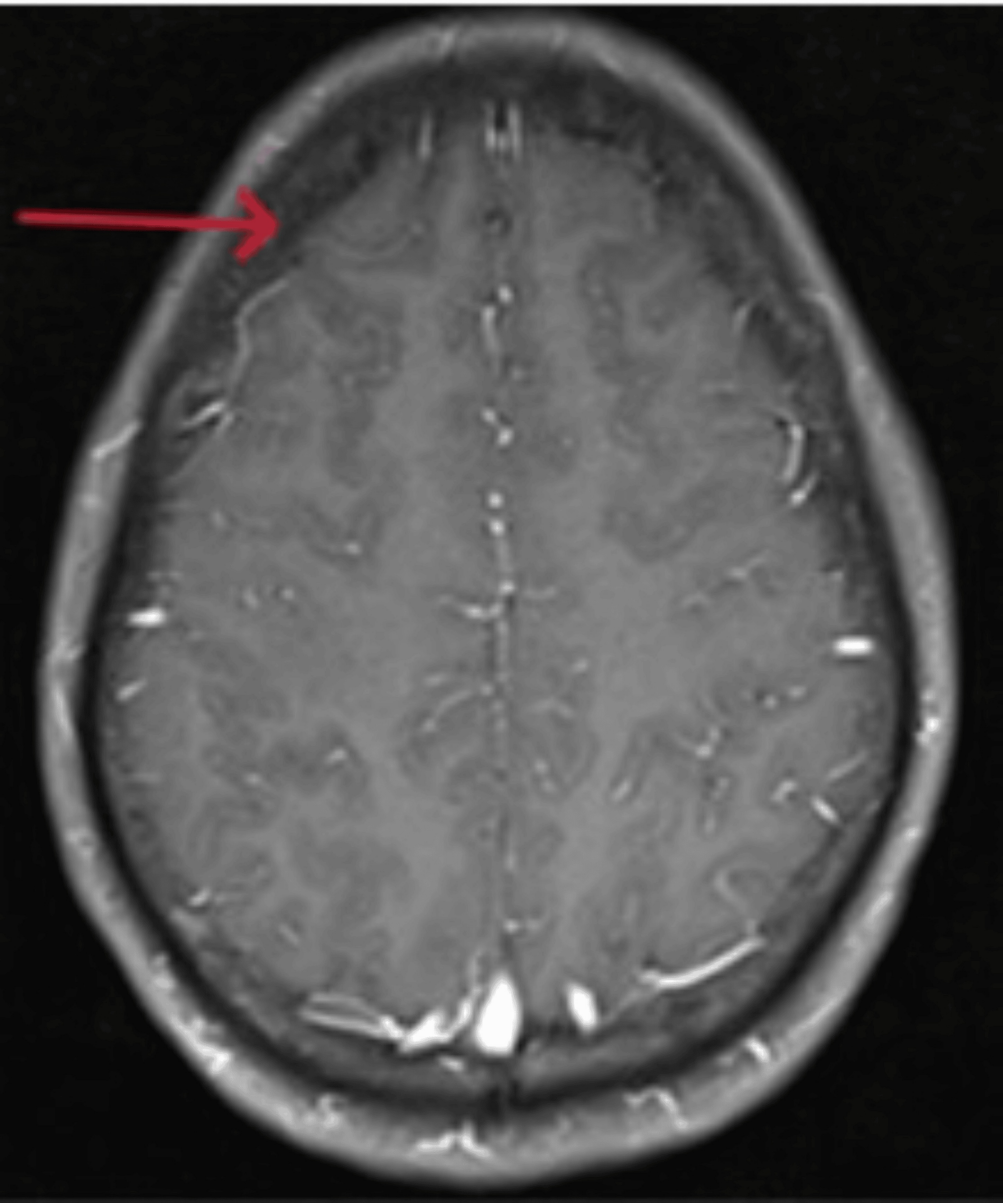
T1 post-contrast showing leptomeningeal enhancement in bilateral high frontoparietal sulci. T1- T1 (longitudinal relaxation time) weighted imaging

**Figure 4:**
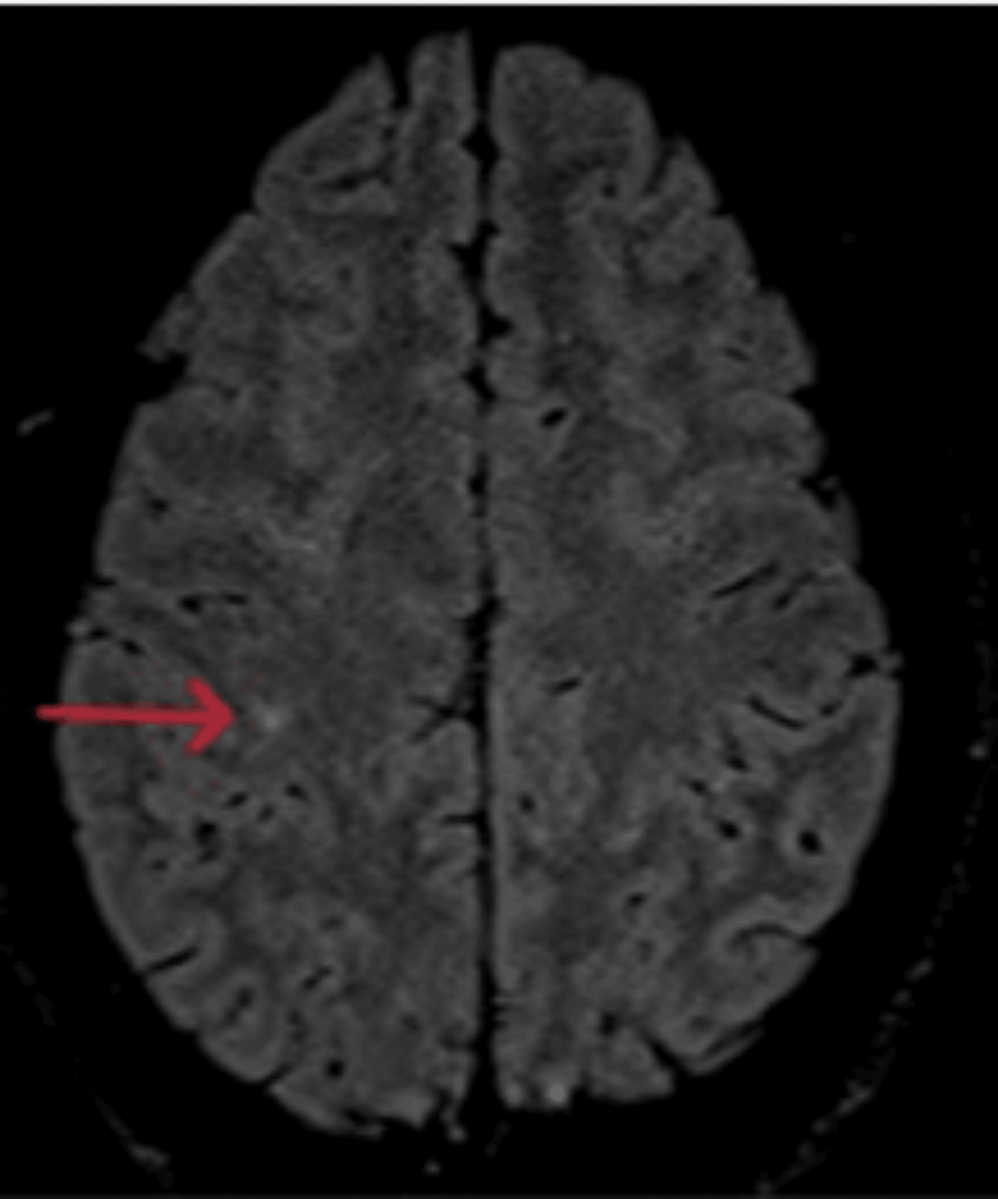
Post-contrast FLAIR showing leptomeningeal enhancement in bilateral high frontoparietal sulci. FLAIR: Fluid-attenuated inversion recovery

On examination, he was tired and irritable. His vitals showed bradycardia and tachypnoea with elevated blood pressure. His body temperature and oxygen saturation were within the normal range. He had no wounds, rashes, prior injuries, lateralizing signs, or signs of meningeal irritation. The cranial nerve examination and fundoscopy findings were regular, and respiratory, gastrointestinal, and cardiovascular system assessments showed no abnormalities. Computed tomography (CT) head (Figures 1, 2) revealed diffuse cerebral edema and sulcal enhancement in bilateral high frontoparietal sulci. Brain magnetic resonance imaging (MRI) with contrast (Figures 3, 4) showed leptomeningeal enhancement in bilateral high frontoparietal sulci. The magnetic resonance (MR) venogram was normal. Laboratory investigations revealed normal blood parameters but a low sodium level (127meq/l). The test results for dengue and malaria infections were negative. The chest X-ray was normal. On the first day of admission, he was empirically started on ceftriaxone (2g twice daily). Other possibilities of infectious etiology were explored with lumbar puncture cerebrospinal fluid (LP-CSF) examination to rule out meningitis. 

**Figure 5:**
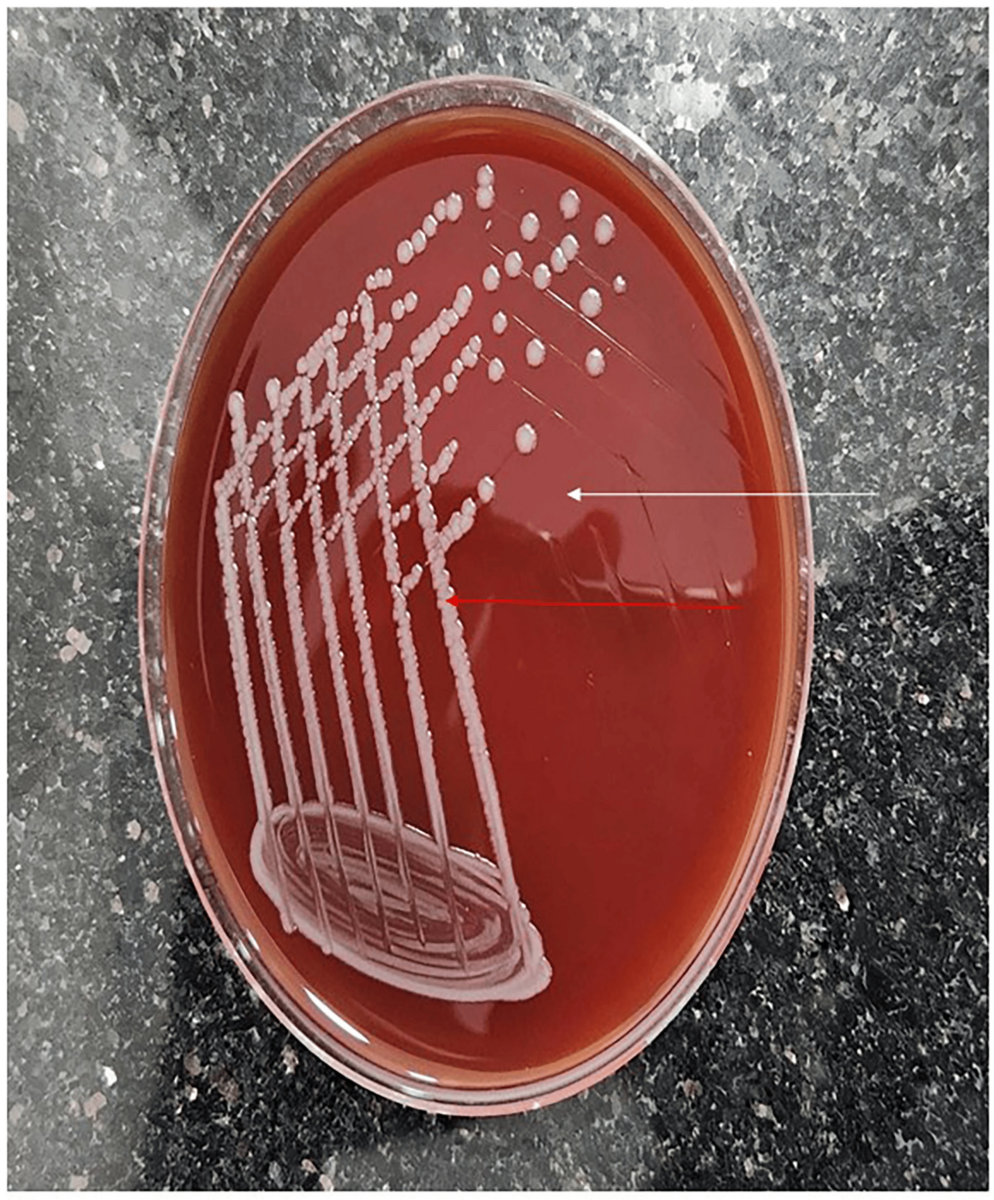
CSF culture isolates (red arrow) against culture medium (white arrow) identified as Cellulosimicrobium cellulans. CSF: Cerebrospinal fluid

On day two of admission, CSF showed neutrophilic pleocytosis with elevated protein and hypoglycorrhachia (Table 1). Gram staining of CSF showed few pus cells, few lymphocytes, and no bacteria. A CSF culture flagged positively after 28h of incubation for a Gram-positive filamentous bacillus- Cellulosimicrobium cellulans (Figure 5). The isolate was susceptible to ceftriaxone, meropenem, gentamicin, and vancomycin.

**Table 1:** Comparison of LP-CSF parameters on admission and after seven days of ceftriaxone. LP-CSF: Lumbar puncture; CSF: Cerebrospinal fluid; WBC: White blood cell; RBC: Red blood cell

Parameter	Day 2 after admission	After seven days of ceftriaxone
CSF colour	Clear	Clear
Protein (mg/L)	286.0	18.9
Glucose (mg/L)	53.0	60.0
WBC count (/cubic mm)	250.0	25.0
RBCs	Absent	Absent
Neutrophils (%)	60.0	0
Lymphocytes (%)	40.0	100.0
Xanthochromia	Absent	Absent

The viral meningitis panel and cartridge-based nucleic acid amplification test (CB-NAAT) were negative. After being admitted to the hospital, the patient suffered fever peaks on days 2 and 3. By the third day of hospitalization, his headache and altered sensorium had improved. The patient recovered significantly and was alert, oriented to time, place, and person, and back to a pre-morbid state. Antibiotics were given intravenously for another week. Adjunct steroids were administered empirically in suspicion of bacterial meningitis. Echocardiography was performed, which showed no evidence of vegetation. A subsequent LP-CSF examination was performed after seven days of ceftriaxone treatment (Table 2). A CSF culture showed no bacterial growth after five days of incubation, indicating the resolution of infection. The patient recovered successfully and was discharged with no neurological consequences. At the two-week follow-up, he was in good health.

**Table 2:** Summary of the case.

Symptoms	Altered sensorium; bradycardia; tachypnoea; elevated blood pressure; afebrile
Lab results	Hyponatremia; blood cultures: negative; CSF parameters: neutrophilic pleocytosis, elevated protein, hypoglycorrhachia; CSF culture: C. cellulans isolated; CSF viral meningitis panel: negative; CSF: cartridge-based nucleic acid amplification test (CB-NAAT): negative
Imaging results	CT head: diffuse cerebral edema and sulcal enhancement in bilateral high frontoparietal sulci; MRI head with contrast: leptomeningeal enhancement in bilateral high frontoparietal sulci; MR venogram: normal; chest X-ray: normal
Treatment	Ceftriaxone: seven days; steroids: Dexamethasone

## Discussion

In this patient, C. cellulans infection caused sporadic adult bacterial meningitis. To our knowledge, at least 43 cases of C. cellulans infections in humans were reported in the literature [3]. According to a recent literature study by Ioannou et al., most infections are seen in men and patients with active malignancies or are immunocompromised, and central nervous system infections account for 5% of all cases reported [3]. In almost all cases, patients have a common feature with a history of indwelling medical devices, or immunosuppression who acquired the C. cellulans infection according to Rivero et al. [2]. Few spontaneous infections have been reported in patients; however, they are rare as discussed by Tucker et al. [4], Kar et al. [5], and Magro-Checa et al. [6]. This case represents the first documented C. cellulans-caused meningitis in an otherwise immunocompetent patient with no similar features.

It is pivotal that though an infection from C. Cellulans is quite rare, we need to note that this organism can cause meningitis in healthy individuals. As we know, certain bacteria cause meningitis, so the possibility of these bacteria should be considered. He had no classic symptoms of meningitis, as he had no fever and no lateralizing signs of meningitis on examination, which makes this diagnosis an important one. 

Another notable feature was the isolation of the bacteria from cerebrospinal fluid which has been noted in the literature where there has been isolation of this bacteria in cerebrospinal fluid in 2.5% of cases [3].

Many cases of C. cellulans infection described treatment with various antibiotics with variable sensitivity among studies mentioned by Rivero et al. [2]. Although there are no standard treatment regimens for C. cellulans infections, many studies recommended vancomycin and rifampicin as the treatment of choice. In our patient, too, the C. cellulans showed sensitivity to vancomycin, but he responded well to ceftriaxone. He was initiated on ceftriaxone as an empirical treatment and it was not changed as he responded very well to the Ceftriaxone. Therefore, the treatment with the latter drug was continued. In patients with C. cellulans infections in individuals with foreign body objects, a prolonged course of therapy with vancomycin is required or requires removal for complete resolution of infection.

Overall mortality was 20% and was attributed directly to the infection in 17.5% [3]. In our case, his infection was resolved, and subsequent CSF cultures were deemed negative for the bacteria and had no neurological sequelae on his follow-up appointment.

## Conclusions

In conclusion, this case of meningitis indicates that* C. cellulans* can infect an immunocompetent person and cause meningitis, mimicking bacterial meningitis caused by other bacterial infections. This case is distinct as this patient did not classically present with features of meningitis. Since there are no standard guidelines for C.cellulans treatment owing to the low number of cases, while ceftriaxone worked for this patient, other studies suggest vancomycin and rifampicin as standard treatments. A follow-up after two weeks showed that the patient was healthy, had no complaints, was well in himself, and had returned to his pre-morbid self. This patient had no similar characteristics to the previous cases reported and is possibly a de novo infection which makes it a novel case by itself.
